# The management of spondyloarthritis in sub-Saharan Africa: a real-world cohort from Kinshasa, Democratic Republic of the Congo

**DOI:** 10.1093/rap/rkag019

**Published:** 2026-01-31

**Authors:** Pierrot Lebughe, Rene Westhovens, Barbara Neerinckx, Jean-Jacques Malemba, Kurt de Vlam

**Affiliations:** Rheumatology Unit, Department of Internal Medicine, Faculty of Medicine, University of Kinshasa, Kinshasa, Democratic Republic of the Congo; Skeletal Biology and Engineering Research Centre, Department of Development and Regeneration, KU Leuven, Leuven, Belgium; Skeletal Biology and Engineering Research Centre, Department of Development and Regeneration, KU Leuven, Leuven, Belgium; Rheumatology Unit, Department of Internal Medicine, Faculty of Medicine, University of Kinshasa, Kinshasa, Democratic Republic of the Congo; Skeletal Biology and Engineering Research Centre, Department of Development and Regeneration, KU Leuven, Leuven, Belgium

**Keywords:** spondyloarthritis, management, DR Congo

## Abstract

**Objectives:**

Data on SpA in sub-Saharan Africa are limited. This study aimed to describe the clinical course and progression of SpA in Congolese patients treated with NSAIDs and physiotherapy and to explore patients’ perceptions of the disease and its treatment.

**Methods:**

This 24-week study involved patients recently diagnosed with SpA and starting treatment over 2 years in the outpatient clinic of the University Hospital of Kinshasa. Demographic and clinical data, including the BASDAI, BASFI and BASMI, were collected at enrolment. All patients were treated with NSAIDs and took part in physical exercise. Assessments were conducted at baseline and weeks 6, 12 and 24. A regression analysis identified factors associated with BASDAI scores >4.

**Results:**

A total of 258 patients were initially enrolled [49% male, mean age 46.5 (s.d. 14.5)]. Of these, 177 completed the study. At baseline, 63.8% had chronic inflammatory low back pain, 42.8% had peripheral arthritis, 46.3% had heel enthesitis and 18.1% had anterior uveitis. At baseline and weeks 6, 12 and 24, the median BASDAI levels were 3.1, 1.9, 0.5 and 0, respectively, and the ASDAS-CRP score declined from 2.1 at baseline to 1.1 at week 24. A total of 37 patients (33.6%) reported a poor perception of their disease. Those with a disease duration of >7 years had greater psychological distress [odds ratio 3.30 (95% CI 1.42, 7.69), *P* = 0.006].

**Conclusions:**

First-line therapy combining NSAIDs and physiotherapy with regular follow-up yielded favourable clinical outcomes in Congolese patients with SpA.

Key messages

- First-line treatment for spondyloarthritis in sub-Saharan Africa is unclear due to a lack of reliable data.
- First-line treatment combining NSAIDs and regular, appropriate physical exercise resulted in satisfaction in terms of reducing disease activity, functional impairment and improving spinal range of motion in Congolese patients.
- High disease activity was associated with greater psychological distress and a duration of symptoms of at least 7 years before the first consultation.

## Introduction

SpA is a group of chronic inflammatory arthritides predominantly affecting the sacroiliac joints and the axial skeleton, often accompanied by peripheral arthritis, enthesitis, dactylitis and extra-articular manifestations [1]. The main symptoms include articular pain and stiffness, reduced spinal mobility and limited range of motion [2, 3]. In severe cases, the patient’s life may be complicated by spinal ankylosis, osteoporosis, infections and an increased cardiovascular risk [4–6]. SpA is classified in two major clinical subtypes: axial SpA (axSpA), including AS and non-radiographic axSpA (nr-axSpA), and peripheral SpA (pSpA), including PsA, reactive arthritis (ReA), arthritis related to IBD and a subtype of JIA [7].

The therapeutic goals are to relieve pain, improve physical function and quality of life, manage extra-articular manifestations and reduce cardiovascular risk [2, 3]. According to the current Assessment of SpondyloArthritis international Society (ASAS)–EULAR and ACR/Spondylitis Association of America/Spondyloarthritis Research and Treatment Network guidelines, first-line treatment includes NSAIDs and structured physical therapy [3, 8, 9]. This combination has been shown to improve clinical outcomes and patients’ quality of life [3, 8–10] as evidenced in several studies and highlighted in systematic reviews [11–13].

Despite the clear stated evidence, data on the clinical course and management of SpA in sub-Saharan Africa (SSA) remain limited. In general, SpA seems to be understudied in this part of the world. Furthermore, the existing literature highlights several relevant issues, particularly those related to diagnosis and clinical presentation [14]. Other relevant issues include access to treatment, especially second-line DMARDs, and continuity of care [15].

Compared with patients in the Western world, patients with SpA in SSA present a milder form of disease activity and functional impairment, a reduced incidence of psoriasis and a lower incidence of family history of SpAs [16, 17]. In the Democratic Republic of the Congo (DR Congo), most patients with SpA are seen in a hospital setting, often at a late stage of the disease. To improve early diagnosis, we recently developed a region-specific screening tool [18] recognizing the different phenotype of the disease in this population. Furthermore, there is an urgent need to identify ways to adapt treatment strategies to local context, particularly focusing on the chronic nature of the disease and the psychosocial impact associated with long-term conditions in a younger population [18–20]. Social barriers are also important, including a lack of acceptance of chronic illness and reluctance towards long-term treatment. Many patients discontinue treatment upon initial symptom improvement despite medical advice. The aim of this study was to evaluate the clinical evolution of SpA in Congolese patients treated with NSAIDs and physical exercise over a 6-month period, as well as to explore their perceptions of the disease and its treatment.

## Methods

### Ethics statement

This research was conducted in accordance with the guidelines of the National Ethics Committee (registration: ESP/CE/154/2020, DR Congo). All participants were informed of the nature, objectives and methodology of this study. Informed consent was obtained in writing prior to inclusion in the study [21]. The data collected were treated confidentially.

### Patients and methods

This study included consecutive patients attending the Rheumatology Department of the University Hospital of Kinshasa for the first time between January 2020 and December 2021. All patients with SpA (axial or peripheral) who met the ASAS classification criteria were included in this study. Participants in a concomitant epidemiological study on SpA conducted in Kinshasa who met the ASAS classification criteria were also included in this study. At the initial consultation, data collected included age, age at symptom onset, sex and pain intensity assessed on a visual analogue scale (VAS), assisted by a rheumatologist if needed. At each visit, a complete clinical examination was performed by a rheumatologist, assessing sacroiliitis, spondylitis, peripheral arthritis, enthesitis, dactylitis and extra-articular features such as uveitis, psoriasis, colitis, gastrointestinal or urogenital infection. Standard X-rays of the pelvis were obtained and sacroiliitis was graded according to the modified New York criteria [22]. Additional radiographs were obtained if clinically indicated. All patients received first-line treatment with NSAIDs and a structured exercise program. They received diclofenac 50 mg orally every 8 h for 4 weeks, followed by on-demand use. Treatment adjustments were made based on clinical evolution and treatment tolerance. Adapted exercise videos were sourced from YouTube [23, 24]. Three physician assistants, trained as co-investigators provided individualized guidance on exercise frequency, rhythm and duration of exercise.

Patients were monitored using the VAS pain, treatment tolerance and self-reported questionnaires assessing disease activity and functional impact of the disease. For patients with axSpA, disease activity was assessed using the French versions of the BASDAI score and the Ankylosing Spondylitis Disease Activity Score with CRP (ASDAS-CRP). The BASDAI and ASDAS-CRP scores were also used to evaluate patients with ReA, given that they have psychometric data supporting their use in pSpA with good discriminatory ability [25, 26]. ASDAS-CRP categories included inactive disease (ASDAS-CRP <1.3), moderate disease activity (ASDAS-CRP 1.3–2.1), high disease activity (ASDAS-CRP 2.1–3.5) or very high disease activity (ASDAS-CRP >3.5). Functional disability was assessed using the BASFI. Spinal mobility was assessed using the BASMI Linear scale for patients with axSpA. Cervical mobility was monitored in patients with ReA. Therapeutic response was evaluated using ASAS 20 response criteria for each appointment. At the end of the evaluation, the ASAS 20 response criteria and the ASAS 40 response criteria were assessed. The rate and the proportion of patients achieving ASAS partial remission were calculated using questionnaires completed by all patients reviewed. All questionnaires were completed with the assistance of trained physician co-investigators. For patients with limited proficiency in French medical terminology, explanations were provided in Lingala. The psychological impact of the disease was assessed using a pre-established four-question survey: ‘Does your illness affect your mind?’, ‘Do you think your illness is of mysterious origin?’, ‘Do you think there could be an alternative curative treatment?’, and ‘Do you think your illness is related to bad luck?’.

Blood tests were performed at baseline and after 24 weeks of treatment and included a full blood count, CRP and creatinine to assess NSAID-related renal toxicity. At baseline, HIV screening was performed according to the national AIDS program guidelines after informed consent. Clinical assessments were carried out at baseline and weeks 6, 12 and 24. Patients who completed both the baseline and follow-up questionnaires received a participation incentive of $5.

### Statistical analysis

All statistical analyses were performed with SPSS version 20 (IBM, Armonk, NY, USA). Descriptive statistics were used to present characteristics: categorical variables as percentages and continuous variables as mean (s.d.) or median [interquartile range (IQR)] as appropriate. The chi-squared test was used for categorical variables and the Mann–Whitney U test for continuous variables. Logistic regression models, including both univariate and multivariate analysis, were used to identify factors associated with a high BASADI at baseline. Odds ratios (ORs) with 95% CIs were reported. *P*-values <0.05 were considered statistically significant.

## Results

**Figure 1:**
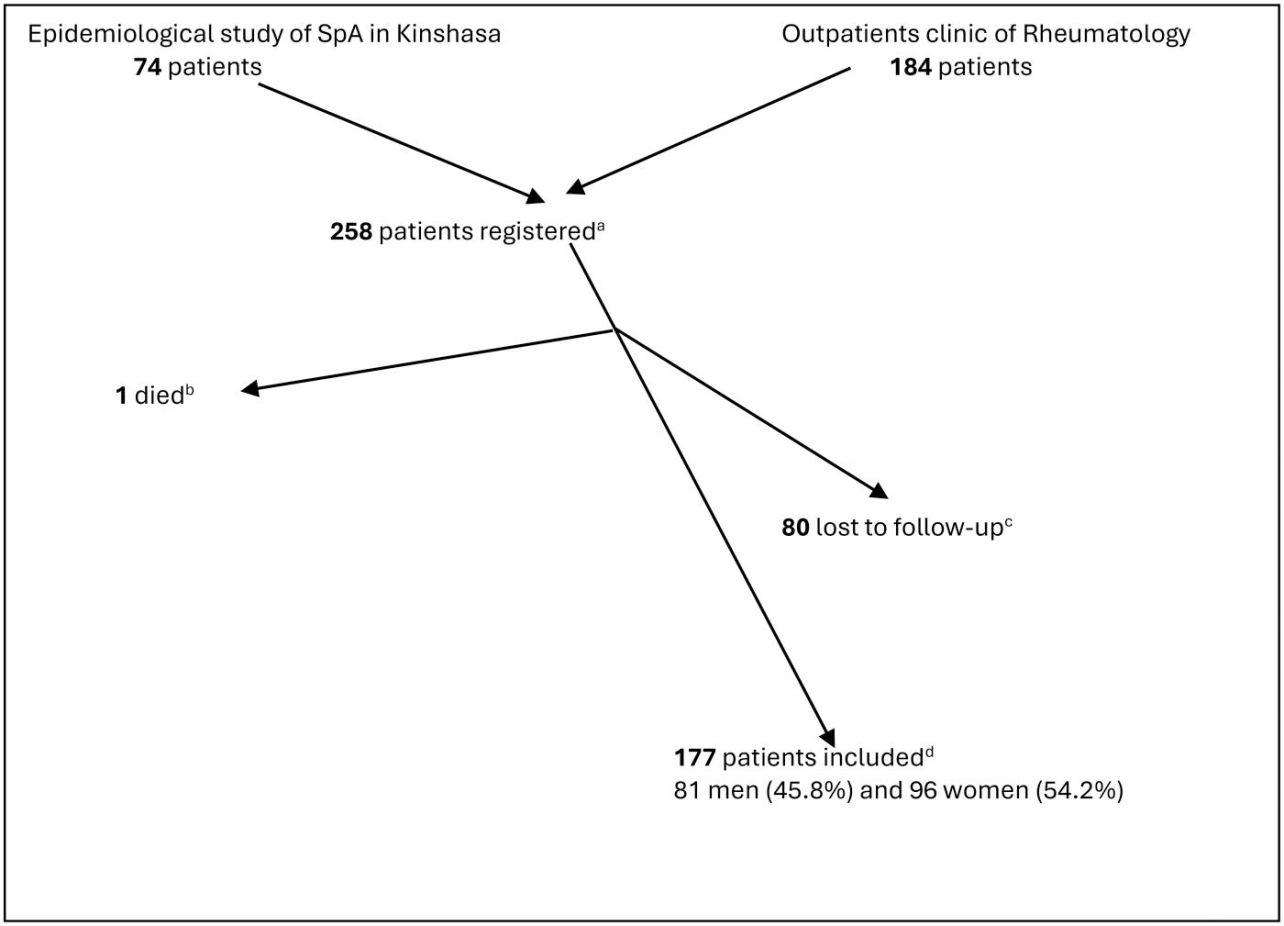
Flow chart. (A) Total number of patients registered at the start of the study. **(B)** Patient who died during the follow-up period. **(C)** Patients lost to follow-up. **(D)** Total number of patients who completed the evaluation at week 24

A total of 258 patients [127 males (49%) and 132 females (51%)] were initially enrolled, 74 from the epidemiological study of SpA in Kinshasa and 184 from the rheumatology outpatient clinic. One patient died without an identified cause. A total of 80 patients were lost to follow-up during the study. The final analysis included 177 patients (96 women and 81 men) (Fig. 1).

The mean age of the patients was 48.4 years (s.d. 10.7) with a mean symptom onset age of 41.7 years (s.d. 12.4). The median delay between symptom onset and first medical visit was 3.1 years (range 2 months–9 years). Table 1 presents the baseline epidemiological and clinical characteristics of the disease. Chronic inflammatory low back pain was the most frequently reported manifestation (63.8%), followed by calcaneal enthesitis (46.3%) and peripheral arthritis (42.8%), and anterior uveitis was the most common extra-articular manifestation (18.1%). AxSpA accounted for 149/258 patients (57.8%), including 54 cases of AS and 95 cases of nr-axSpA. HLA-B27 testing was only performed in 74 patients, including 47 cases of AS, and none were positive. HIV serology was positive in 3/258 patients (1.1%). Of a total of 76 patients with ReA, sacroiliitis was initially found in 15 patients with ReA (19.7%). No patients had coxitis. CRP values in axSpA and pSpA at admission were 6.3 mg/l (s.d. 3.5) and 11.2 mg/l (s.d. 6.8), respectively.

**Table 1:** Sociodemographic and clinical data of patients at inclusion (*N* = 258).

Characteristics^a^	Values
Age, years, mean (s.d.)	46.5 (14.5)
**Age at onset, years, mean (s.d.)**	39.6 (12.1)
Sex, *n* (%)	
Male	127 (49.0)
Female	132 (51.0)
**Disease duration, years, mean (s.d.) (minimum–maximum)**	6.9 (6.8) (0–13)
**VAS pain, mm, mean (s.d.)**	78 (13.5)
Clinical features, *n* (%)	
Inflammatory low back pain	165 (63.9)
Peripheral arthritis	110 (42.6)
Enthesitis/dactylitis	119 (46.1)
Uveitis	47 (18.2)
Psoriasis	3 (1.2)
IBD	2 (0.8)
Diagnostic, *n* (%)	
AS, male	54 (20.9), 34
nr-axSpA, male	95 (36.8), 33
ReA, male	76 (29.5), 39
PsA, male	4 (1.5), 4
SpA IBD, male	1 (0.4), 1
uSpA, male	28 (10.9), 16
**ASDAS-CRP, median (IQR)**	2.7 (1.7–2.8)
BASDAI, median (IQR)	3.4 (2.4–4.5)
BASFI, median (IQR)	2.7 (1.8–3.6)
BASMI, median (IQR)	3.0 (2.5–4.0)
CRP, median (IQR) (normal ≤6 mg/l)	1.5 (0.8–2.5)

uSpA: undifferentiated SpA.

a Data include demographic and clinical characteristics, diagnoses and index assessing disease activity, functional impact and degree of spinal mobility. Disease duration corresponds to the interval between the onset of the first symptoms and the rheumatologist’s diagnosis.

**Figure 2:**
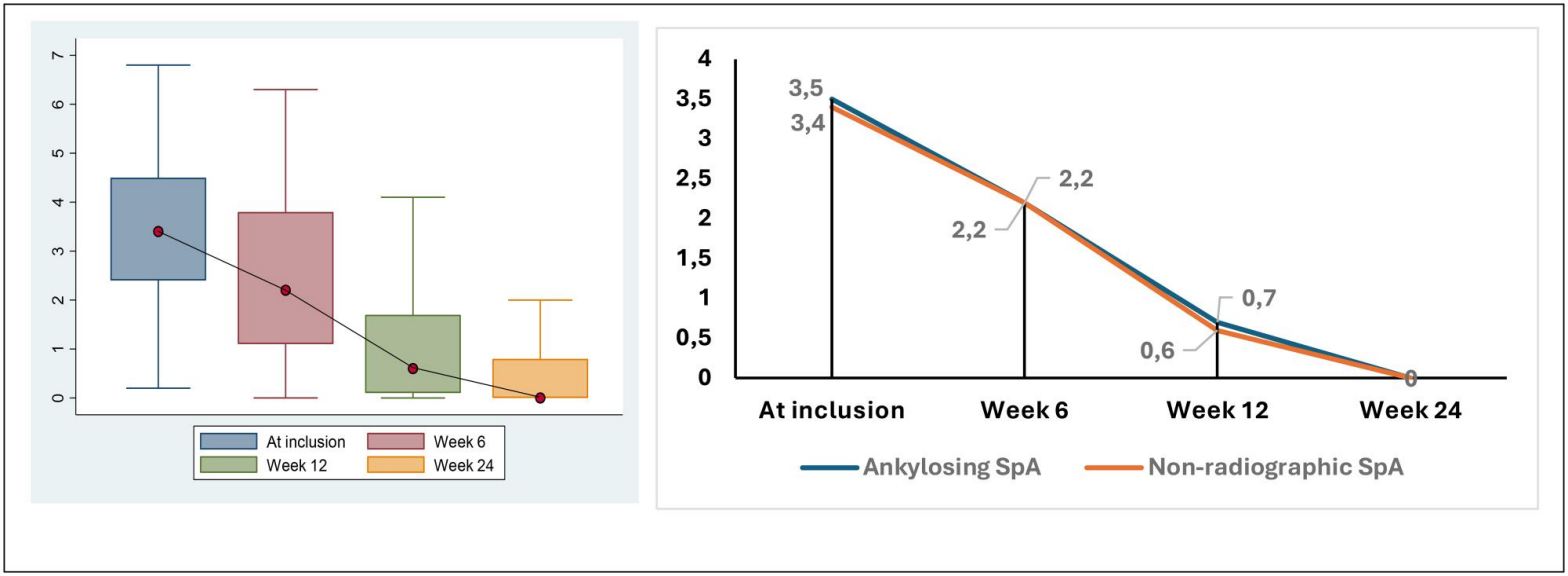
Evolution of BASDAI scores during the study

**Figure 3:**
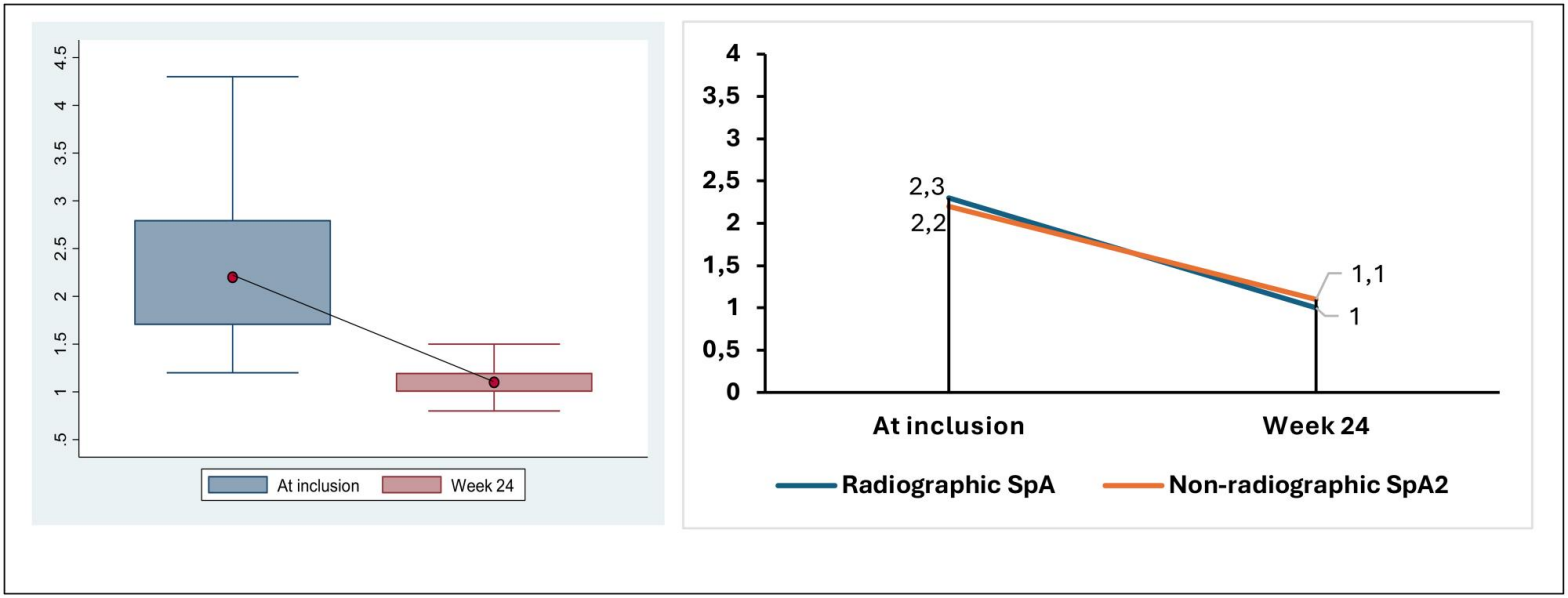
Evolution of the ASDAS-CRP

At week 24, 177 patients had been evaluated, including 119 patients with axSpA (67.2%), comprising 48 cases of AS and 71 cases of nr-axSpA. In patients suffering from axSpA, the evolution of disease activity as measured by BASDAI and ASDAS-CRP over 24 weeks is shown in Figs 1 and 2, respectively. The median BASDAI values at baseline and weeks 6, 12 and 24 were 3.4 (IQR 2.4–4.5), 2.2 (IQR 1.1–3.8), 0.8 (IQR 0–1.4) and 0.4 (IQR 0–0.8), respectively. ASDAS-CRP values were calculated at baseline and the end of the study, with a median score of 2.7 (IQR 1.7–2.8) and 1.1 (IQR 1.0–1.2), respectively (see Fig. 3).

**Figure 4:**
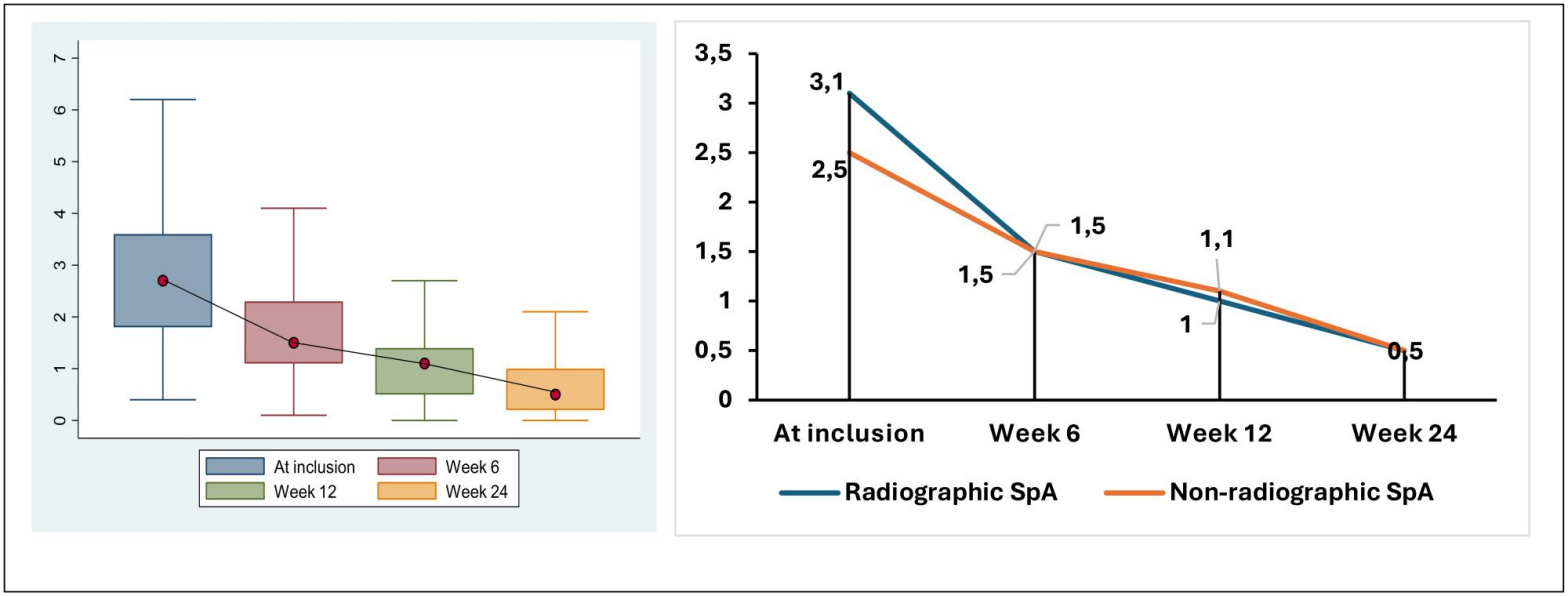
Evolution of BASFI scores during the study

**Figure 5:**
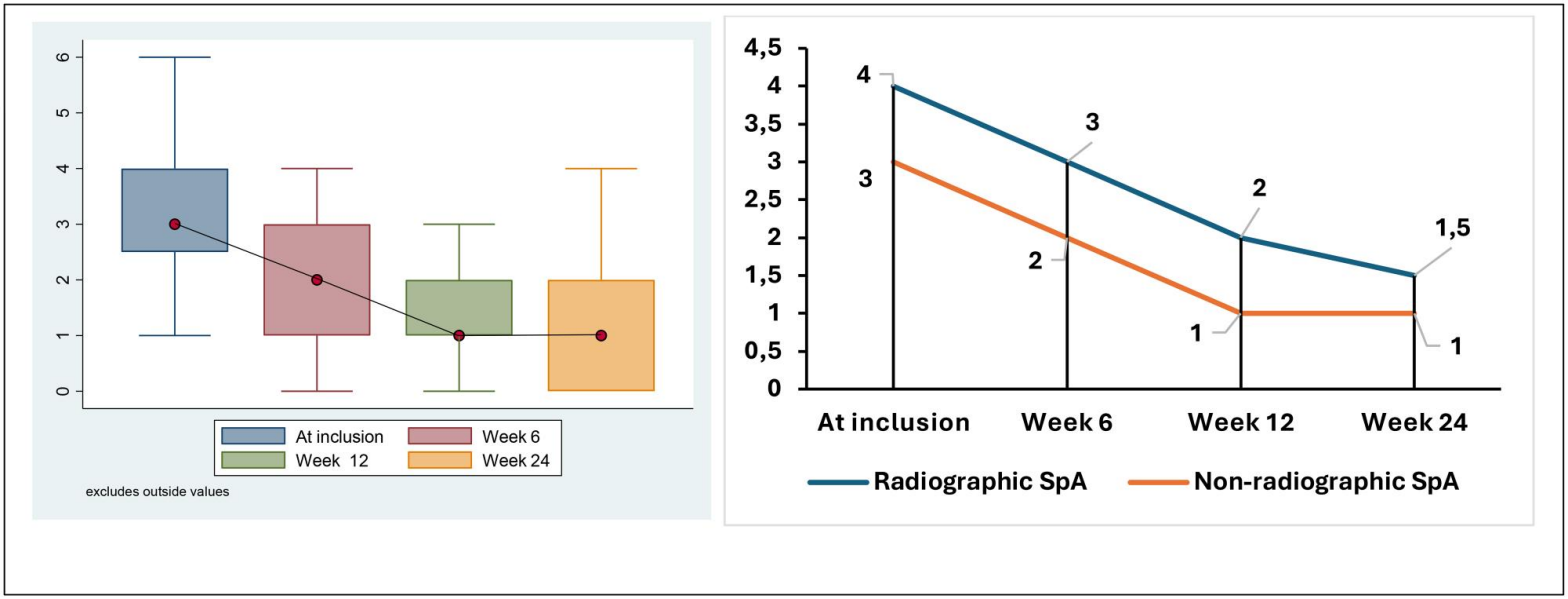
Evolution of BASMI scores during the study

The median BASFI value at admission was 2.7 (IQR 1.8–3.6). The median values at weeks 6, 12 and 24 were 1.5 (IQR 1.1–2.3), 1.1 (IQR 0.5–1.4) and 0.5 (IQR 0.2–1.0), respectively (see Fig. 4). Changes in the median value of the BASMI Linear score at admission was 3.0 (IQR 2.5–4.0). The changes in the BASMI Linear score over time are shown in Fig. 5. Of a total of 76 patients with ReA, only 3 patients (3.9%) had reduced cervical mobility [mean 60° (s.d. 5)] at the final evaluation. Increased disease activity (BASDAI >4 at baseline) was associated with age >55 years and a CRP level ≥6 mg/l but independent for several other variables, including male gender, age at disease onset and time to first visit (Supplementary Table S1).

With regard to disease activity, only three patients (1.7%) had a BASDAI score >4 at week 24. Additionally, 98 patients (82.5%) had an ASDAS-CRP score <1.3 at week 24, indicating inactive disease. Thirteen patients (10.7%) had moderate disease activity while eight patients (6.8%) had high disease activity at week 24. No patient experienced worsening of disease activity throughout the study period. Five patients (4.2%) had a significant improvement, defined as a difference of at least 2 in ASDAS-CRP levels at baseline and week 24. Clinical improvement according to the ASAS 20 response criteria was seen in all patients assessed at week 24, although three patients (2.5%) continued to show high disease activity according to BASDAI and/or ASDAS-CRP scores at the end of the study. The ASAS 40 response rate was 58.0% (69/119 evaluated axSpA patients) and the ASAS partial remission rate was 61.3% (73/119 evaluated axSpA patients) at the end of the study.

A total of 110 patients were surveyed regarding the psychological impact of their illness during the follow-up period. Of these only 43 patients (39.1%) believed that their illness could be cured by traditional medicine (‘Do you think there could be an alternative curative treatment?’), 31 patients (31.8%) recognised that their illness had a negative impact on their mental state (‘Does your disease affect your mind?’), 28 patients (25.5%) reported that their illness was related to a misfortune or unfortunate hazard (‘Do you think your illness is related to bad luck?’) and 9 patients (8.2%) believed in a mysterious origin of their illness (‘Do you think your illness is of mysterious origin?’). Greater psychological distress of the disease was defined as having two or more positive answers out of the four questions. This was observed in 37/110 patients (33.6%). Greater psychological distress of the disease was associated with a disease duration of ≥7 years prior to consultation [OR 3.30 (95% CI 1.42, 7.69), *P* = 0.006]. Patient age >55 years and CRP levels >6 mg/l were associated with a high BASDAI score (>4) on admission [OR 2.35 (95% CI 1.22, 4.52), *P* = 0.010 and OR 4.89 (95% CI 1.77, 13.5), *P* = 0.002, respectively] (Table 2).

## Discussion

This study assessed the progression of patients suffering from SpA undergoing treatment with NSAIDs and physical exercise in Kinshasa, DR Congo. This treatment is the first line of therapy recommended in the guideline [27]. The average level of disease activity, as assessed by BASDAI and ASDAS-CRP scores, was high in the majority of patients at admission. Persistent or exacerbated axial or peripheral pain was indeed the reason for consulting a rheumatologist after several failed treatments, which seems to explain the high activity seen at study inclusion.

The age of the patients and the age of onset were higher than those reported in the Western world. These results are consistent with those reported in the literature in SSA. This phenotype also remains different for African SpA patients living in the Western world compared with Western patients [14–16]. This clinical profile is similar to those found in the Western world in non-HLA-B27 patients, with a late age of onset, few extra-articular manifestations and no family history [19, 28, 29].

This clinical phenotype could result from the interaction of genetic factors other than HLA-B27 associated to a particular environment, notably a rich and probably influential infectious ecology [14, 19, 30]. Several authors in SSA have reported the impact of local infections, particularly HIV, on the epidemiology, clinical features and evolution of SpA, notably in the context of ReA [19, 30–32]. They had more uveitis and less psoriasis and family history of SpA. It is possible that the immunogenetic profile of the patients is different, which could explain the different presentation. The significant influence of self-medication and limited access to the healthcare system could also explain the significant delay between the age of onset and the time of consultation in rheumatology. This long delay could be explained by several factors, including lack of awareness of the disease among healthcare professionals, poor organization of healthcare systems in developing countries, an insufficient number of rheumatologists in the community and a lack of interest in consulting a rheumatologist. The HLA-B27 antigen screening test was negative for 74 of 258 patients tested. This result, although partial, confirms the rarity of HLA-B27 and its weak association with SpA, as reported in our country and in most studies conducted in SSA [17, 33–35]. Risk and protective factors need to be identified in SSA populations to better understand the clinical profile and pathogenesis of SpA. The study showed that the majority of patients suffering from axSpA had achieved clinical remission (82.5%) and no patient had worsened disease activity throughout the monitoring period. Well-managed first-line treatment involving regular medical follow-ups appears to provide satisfactory results in terms of both controlling disease activity and improving function. The clinical profile of SpA tends to be relatively mild, which may favour a positive clinical response in most patients. It is also possible that certain environmental factors, particularly the rarity of smoking in our population, could contribute to a less severe clinical profile with favourable clinical outcomes. The persistent presence of infectious diseases such as malaria, urinary tract infections and digestive infections in our environment could induce persistent stimulation of the immune system, leading to an immunomodulatory effect on the systemic inflammatory response. This could provide an explanation for the low frequency of severe forms. Further studies are needed to verify these hypotheses in SSA. In the same context, a local study by Malemba *et al*. [36] found that the clinical profile of RA was not particularly severe, with a prevalence similar to that reported in Western literature. First-line treatment, particularly low-dose methotrexate, was effective for most patients monitored [37].

Despite the availability of rheumatologists and treatment, the disease’s negative psychological perception remains an obstacle to acceptance. Indeed, the concept of chronicity does not appear to be widely accepted within our cultural traditions [38]. Patients should be informed that their disease is chronic and that the therapeutic goal is to manage symptoms and achieve remission or low disease activity rather than curing the disease. The limitations of this study are the small sample size of patients assessed at the end of the study, the lack of some follow-up data at intermediate stages and the lack of information on HLA-B27 status in the majority of patients. Nevertheless, this is the first study to include a regular follow-up of SpA patients with a rigorous evaluation of a first-line treatment in our setting. The clinical profile seems to be rather mild and regular monitoring of patients seems quite positive. Validation studies of disease assessment tools (BASDAI, BASFI, ASDAS-CRP) should be further conducted in SSA. The definition of clinical remission or low disease activity needs to be agreed upon and validated. Further studies could define the immunogenetic profile of our patients. They could also define the exact influence of environmental factors, in particular the major role of infection in SSA. The notion of chronicity of the disease might be different from that in other regions in the world, and specific assessment criteria for psychological impact and specific education tools should be implemented. Subsequent studies could be conducted to develop the adapted guidelines for the management of SpA in SSA, taking into account the phenotypic profile and socio-economic situations.

## Conclusions

The first-line treatment of SpA using NSAIDs and physical exercise in SSA, especially in the DR Congo, has proven effective. Regular therapeutic follow-up of patients, including psychological support, could ensure an improved clinical prognosis. However, region-specific recommendations are needed.

## Supplementary data

Supplementary data is available at *Rheumatology Advances in Practice* online.

## Supplementary Material

rkag019_Supplementary_Data

## Data Availability

The data used in this study are available upon reasonable request to the corresponding author.
