# Supplementary material for: The management of spondyloarthritis in sub-Saharan Africa: a real-world cohort from Kinshasa, Democratic Republic of the Congo
Source: Rheumatol Adv Pract. 2026 Jan 31;10(2):rkag019. doi: 10.1093/rap/rkag019 (PMC13070704; doi:10.1093/rap/rkag019)
Supplement: rkag019_Supplementary_Data [file rkag019_supplementary_data.zip › 25-186 Supplementary Table S1.docx]

Supplementary Table S1. Factors associated with a BASDAI index greater than 4

| Variables | Univariate analysis^b^ | |  | Multivariate analysis | |
| --- | --- | --- | --- | --- | --- |
|  | OR [IC 95%] | P |  | Adjusted OR [IC 95%] | P |
|  |  |  |  |  |  |
|  |  |  |  |  |  |
| Male | 1.59 [0.55-4.60] | 0.393 |  | 1.75 [0.99-3.11] | 0.055 |
| Age over 55 years | 5.7 [1.27-26.00] | 0.023 |  | 2.35 [1.22-4.52] | 0.010 |
| Delay before consultation ≥ 7 years | 1.19 [1.02-1.40] | 0.028 |  | 1.08 [0.99-1.18] | 0.101 |
| CRP ≥ 6 mg/L | 5.53 [1.57-19.45] | 0.008 |  | 4.89 [1.77-13.5] | 0.002 |
|  |  |  |  |  |  |

a This regression model evaluated four variables, including a strong association with age greater than 55 years at the time of consultation and the presence of biological inflammation. b These variables were evaluated in univariate analysis and then in multivariate analyses to assess the strength of the association (p < 0.05).
